# Radiation-induced myeloid leukemia in murine models

**DOI:** 10.1186/1479-7364-8-13

**Published:** 2014-07-25

**Authors:** Leena Rivina, Michael Davoren, Robert H Schiestl

**Affiliations:** 1 Department of Environmental Health Sciences, University of California, Los Angeles, 650 Charles E. Young Dr. South, CHS 71-295, Los Angeles, CA 90095, USA; 2 Department of Pathology and Laboratory Medicine, University of California, Los Angeles, 650 Charles E. Young Dr. South, CHS 71-295, Los Angeles, CA 90095, USA; 3 Department of Radiation Oncology, University of California, Los Angeles, 650 Charles E. Young Dr. South, CHS 71-295, Los Angeles, CA 90095, USA; 4 JCCC Healthy and At-Risk Populations Program Area, University of California, Los Angeles, 650 Charles E. Young Dr. South, CHS 71-295, Los Angeles, CA 90095, USA

**Keywords:** Radiation carcinogenesis, Leukemia, Animal models, Secondary cancers

## Abstract

The use of radiation therapy is a cornerstone of modern cancer treatment. The number of patients that undergo radiation as a part of their therapy regimen is only increasing every year, but this does not come without cost. As this number increases, so too does the incidence of secondary, radiation-induced neoplasias, creating a need for therapeutic agents targeted specifically towards incidence reduction and treatment of these cancers. Development and efficacy testing of these agents requires not only extensive *in vitro* testing but also a set of reliable animal models to accurately recreate the complex situations of radiation-induced carcinogenesis. As radiation-induced leukemic progression often involves genomic changes such as rearrangements, deletions, and changes in methylation, the laboratory mouse *Mus musculus*, with its fully sequenced genome, is a powerful tool in cancer research. This fact, combined with the molecular and physiological similarities it shares with man and its small size and high rate of breeding in captivity, makes it the most relevant model to use in radiation-induced leukemia research. In this work, we review relevant *M. musculus* inbred and F_1_ hybrid animal models, as well as methods of induction of radiation-induced myeloid leukemia. Associated molecular pathologies are also included.

## Introduction

Cancer diagnosis rates continue to rise as the population of the USA ages. At the same time, post-therapy survival rates are increasing due to advances in medical technology. Over half of the US population will be diagnosed with cancer at some point in their lifetimes, and of these, a further half will receive radiation therapy as part of their treatment regimen [1,2]. Radiotherapy has a number of uses in the modern oncology tool kit. Radiation can be administered as the only part of treatment or more commonly in combination with other treatments such as chemotherapeutic drugs, molecular targeted therapy, or immunotherapy. Outside of cancer treatment, radiotherapy is also routinely used to initiate immune suppression for bone marrow, stem cell, and organ transplantation [3]. However, this widespread use has its risks. The exposure of healthy tissue to radiation as collateral damage from radiotherapy can result in a variety of acute toxicities or chronic secondary malignancies and specifically radiation-induced leukemias [4,5].

Rapid technological advances in radiation oncology have provided a greater degree of targeted radiation delivery to tumor sites, reducing unnecessary exposure of healthy surrounding tissues. This more accurate delivery of radiation has the benefit of increasing maximum tolerated doses and increasing the therapeutic ratio [6,7]. Despite this, the very nature of tumor growth and complex tumor/healthy tissue interaction makes it unfeasible to completely avoid all collateral exposure and therefore all potential subsequent malignancy. This fact calls for the development of alternative biological therapies to supplement technological solutions, in order to reduce secondary toxicity and malignancy risks to the absolute minimum.

Three potential classes of agents could be applied in order to modulate damage to normal tissue. The first class, radiation protectors, consists of agents given prior to radiation exposure. The second, radiation mitigators, would be given post-exposure (PE), but prior to the onset of symptoms, while the third, therapies, would be administered after the onset of symptoms [8]. Only one agent, amifostine [9], is currently approved by the Food and Drug Administration (FDA) to protect normal tissues during irradiation. Amifostine falls only under the first category, with intravenous administration generally occurring a few minutes prior to radiotherapy. In addition, amifostine can lead to toxic epidermal necrolysis and other side effects, making it a less than ideal choice [10]. The government and medical research community recognize that this single therapy is not sufficient. In order to meet this need, the National Cancer Institute (NCI), in collaboration with the National Institute of Allergy and Infectious Diseases (NIAID), has proposed an algorithm to be used in the selection of agents for preclinical and clinical development aimed at decreasing the adverse effects of cancer therapy, including radiation [11]. The use of animal models to validate these agents is a key part of meeting the requirements of this algorithm. Therefore, a comprehensive description of the animal models relevant to the adverse effects of radiotherapy is of great utility to researchers in the field of prospective treatment development. Williams and colleagues have already extensively covered the selection of animal models designed to mitigate and treat the more acute toxicities associated with radiation exposure [12]. The purpose of this work is to provide an updated review of select inbred mouse models for myeloid leukemia.

## Review

### Background

As a mammalian species with a short maturation time, the laboratory mouse *Mus musculus* is one of the best models available for the study of carcinogenesis and its corresponding pathologies. Over time, the laboratory mouse has undergone a significant evolution in its complexity. As researchers continue to delve into its genome and develop precise techniques to manipulate it, it has gained the ability to mimic progressively more precise aspects of the multifaceted disease, that is, cancer. The modern researcher's arsenal contains murine models that range from specific carcinogen-inducible tumors, to xenograft models fully compatible with human neoplastic cells, to humanized mice expressing human genes. Genetically engineered mice (GEM) have now been imbued with the ability to accurately recapitulate the pathophysiological and underlining molecular features of many human cancers [13]. As a result, GEM have replaced many of the genetically homogenous inbred mice once used in environmentally induced cancer studies. With respect to their genetically engineered relatives, older models often developed tumors at low frequencies and with variable latencies. However, GEM specific to a particular question of carcinogenesis are often still difficult to come by, are overly expensive, or have not yet been described to an adequate extent. In addition, as GEM are characteristically designed to follow an exact carcinogenesis progression path, their use precludes the study of alternative mechanisms. Rather, inbred strains allow for genome-wide surveillance for mutations and genome rearrangements, allowing researchers to probe all possible mechanisms. For these reasons, inbred strains remain a cornerstone of *in vivo* cancer research. Despite their flaws, inbred mice have been indispensible in the discoveries of oncogenes and tumor suppressors, as well as the preclinical assessment of the toxic or therapeutic effects of countless agents [14], discoveries critical to the development of GEM.

In this review, we set out to identify inbred mouse models of radiation-induced (RI) cancers, intended for the assessment of efficacy towards interventions aimed at protecting, mitigating, or treating these malignancies. We have concentrated on models specific to myeloid leukemia as this subtype has been identified as one of the most common secondary cancers arising post radiation therapy [5].

### Inclusion criteria

The scope of this review is limited to murine models of radiation-induced myeloid leukemogenesis. It is specifically focused on those cancers designed to induce following exposures to low-linear energy transfer (LET) gamma and X-ray radiations using both high total dose and high dose rate. Carcinogenesis induced from high-LET radiation, genetically engineered mouse models, and xenograft models are outside of the scope of this work. We have also worked to exclude models requiring supplemental treatment in addition to radiation in order to induce carcinogenesis, although we will discuss the results of such treatments where applicable to our models. In order to maximize clinical relevance, we have chosen to focus only on murine models that tightly mimic the underlying molecular pathologies of each type of cancer as observed in humans.

### Radiation-induced leukemia

Leukemia was one of the first cancers recognized as a radiation-induced malignancy in the field of radiation biology. Prior to the introduction of any radiation safety standards, many X-ray workers, mostly physicists and engineers, developed leukemia after working near particle accelerators and other unshielded sources of ionizing radiation. For a dangerously long period, however, the correlation between radiation exposure and leukemia incidence and mortality was merely anecdotal. Significant evidence only began emerging in Life Span Studies following cohorts of Japanese atomic bomb survivors and patients receiving high doses of therapeutic radiation for cervical cancers, tinea capitis, and ankylosing spondylitis [5,15-20]. In a large study, Boice and colleagues established a sharp increase in leukemia incidence following radiation treatment for the uterine cervix carcinoma [21]. Data from the Chernobyl disaster on excess risk estimates of leukemia in adults and children also began to emerge over the last two decades, providing a far more complete data set on age dependence, doses, and latencies [22-25].

Despite vast differences in exposure scenarios, irradiation dose rates and doses, and radiation quality components, the analysis of these studies led to the identification of salient features common to all ionizing radiation (IR)-induced leukemias. In the adult population, acute and chronic myeloid leukemias (AML and CML) are the two most common radiation-induced cancers observed [16,17,19,26-28]. Younger children, exposed between 5 and 9 years of age, appear to be more susceptible to acute lymphocytic leukemia (ALL), while older children are more likely to develop AML. Interestingly, the incidence of chronic lymphocytic leukemia (CLL) does not seem to be influenced by radiation [15]. Leukemia development risk is highest during the first decade following exposure. The risk then decreases over time but never returns to baseline risk [16,17,22,27,29]. Some studies also report sex-specific differences in relative leukemia type and risk [17,19,22,26].

As valuable as epidemiological data is, the use of mouse models alone cannot fully describe radiation-induced leukemogenesis, and it certainly allows no room for the testing of interventions. It is therefore imperative in order to study mechanisms of induction, improve diagnostics, and further the development of radiation protection and mitigation efforts. Multiple established murine models currently exist: RF [30,31], SJL/J [32], CBA [33,34], and C3H/He [35]. Table 1 summarizes the optimal induction method and associated myelogenous leukemia (ML) frequencies.

**Table 1:** Induction of myeloid leukemia in mice with low-LET ionizing radiation

**Mouse strain**	**Age (weeks)**	**Sex**	**Dosage (Gy)**	**Fractionation**	**Latency (months)**	**Spontaneous frequency (%)**	**Induced frequency (%)**	**Reference**
RF (RF/J, RFM)	8	M	4.25	Single	4–12	2–4	50–90	[30,41]
SJL/J	8–10	F	3–3.5	Single	12	0	10–30	[31]
C3H/He	8–10	M	2.84	Single	1.5–18	<1	25	[32]
CBA (CBA/Ca, CBA/Cne, CBA/H)	12–15	M	3	Single	18–24	0.1–1	25	[34]

### RF mouse

The RF mouse was developed as a general-purpose stock from A, R, and S strains at the Rockefeller Institute [31,36,37]. Its propensity for radiation leukemogenesis has been studied extensively by Upton and colleagues [38]. Detonation experiments conducted by Furth and colleagues in 1936 provide one of the earliest accounts of leukemogenesis in this strain [39]. In the RF model, ML is induced with a single dose of ionizing radiation. This has been proposed as a counterpart to human AML, particularly due to the diagnosable tissue lesions present during a prolonged preclinical period [31].

At 18–24 months of age, a 2%–4% background incidence of myeloid leukemia is observable in RF mice [40]. Exposure of 8-week-old RF males to 1.5 Gy increases lifetime ML incidence to about 40%, while *in utero* and neonatal exposures paradoxically *decrease* ML induction [30,41]. Dosing these males at 4.25-Gy ML increases incidence to 50%–90%, with a latency period of 4–6 months [31,38,42]. An enlarged spleen and liver can be seen to accumulate young myeloid cells from as early as 12 weeks post exposure. Clinically, leukemia in RF mice presents with infiltration of peribronchial areas, lymph nodes, and gastrointestinal lymphoid organs. However, at the dose necessary to induce ML, the rate of thymic lymphoma induction also increases to about 25%, potentially interfering with accurate ML diagnosis and confounding modeling of the human disease [31]. A sex difference in susceptibility to thymic lymphoma (TL) and ML was also demonstrated by Upton et al. RF females are more susceptible to TL, while male mice are more likely to develop ML [30].

Hayata et al. have reported that myeloid leukemia in the RF model exhibits partial deletion of chromosome 2, along with other genomic instabilities and loss of the Y chromosome [43], in a manner similar to radiation-induced leukemia in the SJL/J mouse [44]. The protracted latency of ML in RF mice correlates well with human data. The peak incidence of leukemia occurred 5–10 years post exposure in both Japanese atomic bomb survivors and children exposed to the Chernobyl disaster, corresponding well with mouse latency [17,22,26,45]. However, the RF mouse model's utility is limited by its propensity to present with mixed hematopoietic tumors of myeloid leukemia and thymic lymphoma [30].

### SJL/J mouse

The SJL/J strain, developed by Murphy in the 1960s, is known for its high spontaneous frequency of reticulum cell neoplasms (type B, RCN B) [46,47] occurring roughly 380 days after birth in both males and females. As the histological pattern of these RCNs presents similarly to that of human Hodgkin's disease, this strain has been proposed as an investigative model for this cancer [48].

A single, whole-body exposure of 8–10-week-old female SJL/J mice to 3.0–3.5 Gy induces myeloid leukemia in only 10%–30% of treated animals within a year. However, Haran-Ghera et al. have also observed that exposure to fractionated X-rays induces lymphosarcomas [48]. Consistent with AML diagnosis, leukemic infiltrations are observed in the bone marrow, lymph nodes, spleen, and liver, consistent with a diagnosis of AML [32]. The frequency of developing radiation-induced acute myeloid leukemia (RI-AML) increases with the age at radiation exposure up to 12 weeks. It has been proposed that this increase in susceptibility is explained by the development of the mouse's mononuclear phagocytic system [49].

While radiation is sufficient to initiate RI-AML, this complex, multiphase malignancy often requires the administration of additional promoting factors in order to fully recapitulate tumor development [50]. Preleukemic cells, as well as the characteristic chromosome 2 deletions described previously, are observed in the bone marrow of the overwhelming majority of IR-treated mice, prior to the clinical presentation of overt AML at 90–120 days [51,52]. However, boosting the relatively low radiation-only induction rate requires the administration of corticosteroids following irradiation. This increases RI-AML incidence to 50%–70% [32]. Coadministration of growth factors, especially colony-stimulating factor-1 (CSF-1), decreases latency and increases frequency even further to 75% [50,53]. The significance of this particular factor is supported by the fact that, 2–4 months prior to RI-AML onset, those 10%–30% of RJL/L mice that will develop solely radiation-induced cancer have significantly elevated CSF-1 levels as compared to those mice in which RI-AML fails to develop or those that develop RCN B. The observation that RI-AML cells *in vitro* synthesize significant amounts of CSF-1 further supports the hypothesis that CSF-1 is necessary for leukemia progression [49].

The clinical presentation of RI-AML in the SJL/J mouse closely resembles that of secondary leukemias observed in man [32]. The development of AML has been reported at high frequencies in Hodgkin's disease patients in remission after radiation treatment and steroid regimens [54,55]. This correlation between a Hodgkin's disease/RCN B background state and the induction of RI-AML afterwards makes SJL/J an extremely valid RI-AML model. These mice only develop the AML type of leukemia, similar to irradiated Hodgkin's disease patients [56]. Elevated circulating levels of CSF-1 have also been reported in some neoplastic malignancies, including AML, and appear to be associated with poor prognosis [57-60], further promoting the use of the SJL/J mouse in the study of the CSF-1's role in cancer.

### C3H mouse

The venerable C3H strain was developed by Strong in 1920, from a cross of the Bragg albino mouse and the DBA mouse. Strong generated this strain while specifically selecting for the elevated incidence of mammary tumors (MT). Ninety percent of unfostered pups, those pups remaining with their birth mother *postpartum*, develop mammary tumors by 11 months of age due to transfer of mammary tumor virus (MTV) from the mother's lactation. Fostering the offspring or transferring fertilized ova to a mammary tumor virus-free surrogate significantly reduces tumor development frequency [36,37]. However, the fostered C3H/He substrain has a high incidence of spontaneous hepatomas later in life [35,61].

Three gray of whole-body X-irradiation in 8–10-week-old male C3H/He mice induces myeloid leukemia in 23.9% of exposed animals, with myelomonocytic leukemia being the most prevalent subtype. Dose-response curves in C3H mice are similar to those in RFM and CBA mice, with a proportional increased leukemia induction frequency until a critical dose of around 3 Gy, after which point the incidence rapidly drops off [33]. Yoshida et al. have also reported significant sex differences with females being less susceptible to RI-ML. Similarly to steroid-based promotion in SJL/J mice, the administration of the synthetic glucocorticoid prednisolone following irradiation of C3H/He mice increases the incidence of ML to 38.5% [32]. Suppression and promotion of hematopoietic recovery is suspected as the mechanism of induction. Spontaneous incidence of leukemia is less than 1% [35]; however, this rate can be entirely eliminated by reducing the daily caloric intake to about two thirds of the normal level. Interestingly, the incidence of RI-ML can also be decreased to 7.9% when restriction is started before 6 weeks of age or to 10.7% when restriction is started post radiation exposure at 10 weeks of age [62]. Caloric restriction has also been observed to promote PE longevity via insulin pathway modulation [63]. Chronic inflammation may also be implicated as an exacerbating factor in the promotion of leukemogenesis. Yoshida et al. demonstrated that the induction of chronic low-level inflammation by insertion of a cellulose acetate membrane increases RI-ML incidence to 35.9% [64].

In the C3H/He strain, the partial deletion of chromosome 2 has been implicated in RI-AML development, just as in RFM and SJL/J mice [43,65]. During the first metaphase PE, as little as 24 h after irradiation, chromosome 2 deletions can be detected in the bone marrow of the C3H/He mouse, suggesting that chromosome 2 deletions act in the initiation stages of leukemogenesis [66]. The Ph^1^ chromosome transformation, common in human chronic myeloid leukemia, can be compared to these murine chromosome 2 aberrations in both incidence and disease specificity [67,68].

### CBA mouse

The CBA mouse, also developed by Strong in 1920, is a cross between a Bragg albino female and a DBA male, but selecting for a low mammary tumor incidence. In the CBA/Ca substrain, males tend to have a shorter lifespan than their female counterparts [36,37]. Both the CBA/Ca and CBA/H substrains are directly descended from the original CBA mouse derived in the UK [69,70].

A 3-Gy gamma or X-ray total-body irradiation of 12-week-old male CBA/H mice results in a 25% rate of myeloid leukemia induction. This leukemia infiltrates the sternal bone marrow, liver, and spleen, which serves as a diagnostic endpoint [33,34]. The dose-response curve of leukemia induction is curvilinear, implying a threshold dose as in the models previously discussed. The fact that leukemia is rarely observed in cases with high exposure correlates with human epidemiological data [71,72].

Chromosome 2 (Chr2) aberrations have been noted in these mice and correlated with myeloid leukemia development, just as in the other models [70,73,74]. The expansion of cells carrying Chr2 lesions is present in 20%–25% of irradiated mice and can be observed from as early as 20 h PE to as late as 24 months [75]. Bouffler et al., however, were not able to conclusively prove that the induction and presence of an aberrant Chr2 clone can accurately predict development of RI-AML in CBA mice [76]. Aberrations on chromosome 4 were also reported in about 50% of CBA/H mice diagnosed with typical AML. *Lyr2*/TLSR5 allelic loss was identified as a likely event in radiation-induced hematopoietic malignancies, including myeloid and lymphoid mouse leukemias, by Cleary et al. [77]. An 8% decrease in DNA methylation, not observed in AML-resistant C57Bl/6, has also been linked to RI-AML susceptibility in the CBA/H strain [78].

The CBA mouse is presently the favored RI-AML model for human AML, for three main reasons. It has a low spontaneous frequency of AML, has a mean latency of 18 months, and closely resembles the human malignancy in terms of morphology [69,79]. In addition, Dekkers et al. have suggested that the two-step mutation model of RI-AML in CBA/H, as extrapolated from X-ray and neutron exposure data, is useful in modeling human RI-AML [80].

### ML-associated molecular pathologies

As been discussed relative to the previously mentioned strains, anomalies involving chromosome 2 in particular are closely linked to the development of AML in the mouse model (RF, C3H/He, CBA, and SJL/J) [43,44,65]. Rodents have had particularly high levels of chromosomal recombination over evolutionary time, so determining the directly corresponding human chromosome for a particular mouse segment is often a complex task [81]. Amongst the genes present on mouse Chr2 is the *Abl* gene, found on Chr9 in humans and famous for its fusion into the Bcr-Abl fusion protein in the Philadelphia chromosome. The Philadelphia chromosome is usually associated with CML, and it can also be found in ALL and other leukemic lineages [82,83]. Although aberrant activation of this gene should be considered, other sources of Chr2-based oncogenesis are more likely. As the prototypical Chr2 aberration was best defined as a deletion, the loss of a tumor-suppressing function was identified as a more likely scenario in the oncogenesis process than activation of an oncogene [84]. In 2004, Cook and colleagues identified the *Sfpi1* gene, encoding the transcription factor PU.1, in the 2-Mbp region commonly found deleted from Chr2 in AML [85], after having previously established its general location as a common region of loss of heterozygosity (LOH) [84,86].

The *Sfpi1* gene is a key factor in normal hematopoiesis, involved in the promotion, differentiation, and regulation of every hematopoietic lineage. It is essential for proper terminal myeloid cell differentiation (macrophage and neutrophil), as well as stem cell maintenance [87-91]. Normally, lower levels of PU.1 lead to lymphocyte fates, while higher levels lead to myeloid fates in developing hematopoietic cells, although proper function is required for successful development in both cases [88,92]. PU.1 function is critical for leukemic transformations in mouse myeloid cells. However, its importance in equivalent human transformations is still a subject of active debate [85,93,94]. The PU.1 protein contains DNA-binding and protein-protein-interacting domains. The presence of regulatory phosphorylation sites is imperative for its function [95].

After loss of one copy via deletion of its local region from Chr2, the second copy of *Sfpi1* is often inactivated via point mutations in its DNA-binding region [85,93]. Homozygous conditional knockdown of PU.1, leading to expression levels at about 20% of wild type, induces AML in mice inactivated from birth by 3–8 months of age [96]. Myeloid leukemia is also induced when inactivated in adult mice [97]. The loss of the genomic region coding for PU.1 is a common ‘second hit’ leukemogenesis event in transgenic mice already expressing the oncoprotein PML-PAR [98]. Upregulation of *c-myc* has also been reported accompanying PU.1 deficiencies in AML cells [99]. Forced expression of PU.1 at WT levels in promyelocytic leukemia cells was demonstrated to inhibit clonogenic growth, force monocytic differentiation, and induce apoptosis by Cook et al. These findings support the hypothesis that the suboptimal expression of PU.1 can be a key event in the permission of leukemogenesis by blocking proper maturation of the cell [85,91]. Peng et al. have suggested the quantification of PU.1-deleted bone marrow cells as a surrogate marker for RI-AML [100].

Given these data, it would be tempting to declare PU.1 a tumor suppressor. However, other studies have shown that *over*expression of the very same transcription factor can lead to other cancers, in particular erythroleukemias [101]. It would be more correct to argue that PU.1 is a critical transcription factor involved in the differentiation of multiple hematopoietic lineages, the dysregulation of which serves the development of many leukemic variants.

The human ortholog of PU.1 exists on chromosome 11 [91] and is expressed at low levels in most AML cases, as might be predicted from the mouse models [102]. However, inactivation by deletion of *SPI1* is comparatively rare in man [93,94]. Cook et al. proposed that other mechanisms of PU.1 deactivation take precedence in human AML: the gene could be epigenetically silenced or inactivated through interaction with a mutated receptor (i.e., Flt3 cytokine receptor that is found in 25% of human AML) or another protein [85]. The aberrant expression of certain miRNAs, specifically miR-155, has also been suggested as a cause of reduced PU.1 expression [103]. Interestingly, Finnon et al*.* recently showed that the *Flt3*-ITD and *Sfpi1*/PU.1 mutations are mutually exclusive in murine radiation-induced AML, without any overt phenotypic differences, suggesting that the two are capable of playing an equivalent role in the oncogenesis process [104]. The group did not report on the actual levels of PU.1, so it remains plausible that PU.1 depression is still involved in these RI-AMLs.

It remains to be tested whether radiation is usually responsible for only one or both of the genomic events commonly observed in RI-AML. Deletion of *Sfpi1* on Chr2 results in the mutation of the PU.1 DNA-binding domain. It is suggested by present data that IR induces the Chr2 deletions [52,65,100], but whether the deletion results from direct DNA damage or from delayed genomic instability remains to be proven [105-107]. In the case of the direct alteration of the *Sfpi1* allele seen in RI-AML cells, however, radiation is not the most likely candidate, as IR does not induce the point mutations observed in *Sfpi1 *[85,93,99]. Evidence suggests that these mutations are of spontaneous origin, as point mutations are the most common of this type [108,109].

Ban and Kai have demonstrated that hematopoietic stem cells (HSCs) surviving 3-Gy radiation are subjected to replicative stress, contributing to accelerated senescence. This decreases replicative fidelity and increases the rate of mutation accumulation presumably including point mutations in the remaining copy of the *Sfpi1* gene. Mathematical models fitted to experimental data from cobblestone area-forming cells (CAFC) and colony-forming unit-granulocyte/macrophages (CFU-G/M) on *ex vivo* bone marrows revealed that irradiated HSCs cycle as much as ten times more than those from unexposed animals [109].

The commonly accepted paradigm that HSCs are the target cells of RI-AML was recently challenged by Hirouchi et al. Instead, they concluded that AML stem cells can arise from long-lived HSCs, short-lived multipotent progenitors (MPPs), and even common myeloid progenitors (CMPs) that have acquired self-renewal potential, with the inactivation of *Dusp2* on Chr2 being a likely contributor. The cell surface phenotypes and gene expression profiles of AML stem cells in their study very closely resembled normal CMPs instead of HSCs [110].

In addition to the relevant Chr2 regions discussed above, loci on Chr8, Chr13, and Chr18 have been identified as involved in leukemogenesis. On Chr18, the gene *Rbbp8*, encoding CtIP, is upregulated in response to X-ray exposure in RI-AML-sensitive CBA mice but not the RI-AML-resistant strain C57BL/6. The human ortholog RBBP8 is a suspected tumor suppressor found on our own chromosome 18. Deletions of *Rbbp8* have been identified in many cancers including AML [111].

## Conclusions

The ideal radiation-induced carcinogenesis mouse model possesses a low spontaneous background frequency of the desired malignancy, has a short latency period, does not develop any other cancers besides the one to be studied, and produces tumors nearly identical to the corresponding human cancer in onset, progression, and underlying pathology. As a perfect model does not exist, researchers are inevitably forced to compromise on some of these features. It is generally more feasible to compromise on features such as cancer latency and induction frequency, as these can be compensated for by study design and sheer subject volume. However, one cannot compromise on the accurate emulation of molecular and pathophysiological features of human radiation-induced malignancies, as these are the features that make a model relevant in the first place. More advances must be made towards the development of more accurate recapitulations of human radiation-induced cancers. Radiation-induced secondary cancers can still be difficult to discern from primary tumors in humans due to unresolved questions about their respective molecular signatures. Identifying and investigating these signatures in mouse tumors following IR is a difficult challenge but brings great potential reward.

The field of radiation mitigation with respect to reducing cancer rates in exposed individuals is still developing, with a few promising developments. Administration of antioxidants appears to reduce the damage absorbed from irradiation. Kuefner et al. observed a significant reduction of H2AX foci, markers of DNA damage, upon *in vitro* preincubation of human lymphocytes with glutathione before irradiation, but this effect did not extend to post-irradiation incubation, nor is it clear whether this effect might carry over to *in vivo* experimentation [112]. As mentioned previously, amifostine and its active metabolite, WR-1065, have been shown to have some promise differentially protecting healthy tissue during radiotherapy when administered beforehand [9,113]. The use of other micronutrients, such as DNA cofactors and selenium, has also been suggested [114]. No clear agent stands out yet, however, as the perfect agent to protect against both radiation-induced toxicity and subsequent cancer risk. As with all complex drug/disease interactions, the use of mouse models to determine an effective treatment is an imperative. If a compound can be conclusively shown to effect the myeloid leukemia rates in these establish models, it would have an extraordinary impact on the field of oncology.

This review presents an updated discussion of the array of myeloid leukemia mouse models. The mouse models presented are often a compromise on the background frequencies and rates of induction, but all demonstrate strong molecular and phenotypic correlations to salient features of the human cancers they are meant to represent. These models provide a powerful tool for testing the therapeutic benefit of candidate drugs against radiation-induced carcinogenesis.

## Abbreviations

ALL: acute lymphoblastic leukemia; AML: acute myeloid leukemia; CAFC: cobblestone area-forming cells; CFU-G/M: colony-forming unit-granulocyte/macrophages; CLL: chronic lymphocytic leukemia; CML: chronic myeloid leukemia; CMP: common myeloid progenitor; GEM: genetically engineered mice; HSC: hematopoietic stem cell; LET: linear energy transfer; MPP: multipotent progenitor cell; MT: mammary tumors; PE: post-exposure; RCN: reticulum cell neoplasms; RI: radiation-induced.

## Competing interests

Robert H. Schiestl is involved in RadMit, Inc. The other authors declare that they have no competing interests.

## Authors’ contributions

LR researched the mouse models in use for a wide variety of radiation-induced cancers, compiled articles describing the relevant molecular pathologies, and drafted the manuscript in its initial form. MD participated in manuscript rewriting and reorganization, added focus on the use of these models in developing radiation mitigators, and compiled additional articles describing further relevant details. RS participated in the grand design of the article, rewrote and pared down to focus specifically on radiation-induced leukemia, and used his expertise in the field to lead to additional areas of research and inquiry. All authors read and approved the final manuscript.
